# Substrate Utilization by the Failing Human Heart by Direct Quantification Using Arterio-Venous Blood Sampling

**DOI:** 10.1371/journal.pone.0007533

**Published:** 2009-10-21

**Authors:** Junichi Funada, Tim R. Betts, Leanne Hodson, Sandy M. Humphreys, Jon Timperley, Keith N. Frayn, Fredrik Karpe

**Affiliations:** 1 The Oxford Centre for Diabetes, Endocrinology and Metabolism, Churchill Hospital, Oxford, United Kingdom; 2 The Department of Cardiology, John Radcliffe Hospital, Oxford, United Kingdom; 3 NIHR Oxford Biomedical Research Centre, Oxford Radcliffe Hospital Trust, Oxford, United Kingdom; Innsbruck Medical University, Austria

## Abstract

Metabolic substrate utilization of the human failing heart is an area of controversy. The purpose of this study is to directly quantify myocardial substrate utilization in moderately severe heart failure, type 2 diabetes and healthy controls using simultaneous coronary sinus and arterial blood sampling. Patients with heart failure (n = 9, mean NYHA 2.7±0.5), with type 2 diabetes (n = 5) and with normal heart function (n = 10) were studied after an overnight fast in connection with electrophysiological investigations/treatments.

A systemic infusion of [^2^H_2_]palmitate allowed for the calculation of absolute palmitate extraction across the heart. Blood samples were analysed for non-esterified fatty acids, triacylglycerol, glycerol, glucose, pyruvate, lactate, 3-hydroxybutyrate, and blood gases after simultaneous sampling of arterial and coronary sinus blood. Arterio-coronary sinus metabolite concentration differences and fractional extractions for all substrates were similar between the groups. The absolute NEFA uptakes assessed by [^2^H_2_]palmitate extraction were also similar between the groups. Using direct measurements of metabolic substrate uptake by arterio-venous difference technique, the compensated human failing heart does not appear to have reduced myocardial fatty acid uptake.

## Introduction

The healthy human myocardium displays a phenomenal ability to switch from fatty acid utilization in the fasted state to glucose utilization in response to insulin [1]. However, if glucose utilization is forced this enhances myocardial energy efficiency is demonstrated; not only is less oxygen consumed per ATP generated, the mechanical output for a given quantity of oxygen consumption is also increased [2].

Substrate utilization of the failing heart has recently been reviewed [3]. Abundant data suggest that rodent models of heart failure show increased myocardial glucose dependence whereas the literature is conflicting for dogs and humans. Also, patterns of gene expression would support a preference towards glucose utilization in failing compared with normal human hearts [4]. Whether a shift towards glucose utilization is an adaptation to more efficiently generate ATP, or a maladaption with restrictions in the flexibility of substrate utilization, is still unclear [5].

The evidence for a shift towards glucose utilization in human heart failure arises from non-invasive monitoring using PET imaging of labelled glucose and palmitic acid moieties. Davila-Roman and colleagues [6] compared substrate utilization in controls and patients with idiopathic dilated cardiomyopathy after serial infusion of ^15^O-water (blood flow), ^11^C-acetate (myocardial oxygen consumption), ^11^C-glucose (myocardial glucose uptake) and ^11^C-palmitic acid (myocardial fatty acid uptake and oxidation) finding a shift away from fatty acid uptake/oxidation towards glucose uptake in failing hearts. An earlier study by Taylor et al. [7] compared non-metabolizable glucose (^18^F-deoxyglucose) and palmitic acid (^18^F-6-thia-heptadecanoic acid) with a conclusion essentially contradictory to Davila-Roman et al. [6], but this study lacks matched controls. Also, Wallhaus and colleagues have used non-metabolizable substrates in a study of heart failure in which carvedilol was used therapeutically to improve cardiac function [8]. They observed a reduction in fatty acid uptake in combination with a non-significant increase in glucose uptake as heart function improved. The reason for these conflicting results is not clear but it could depend on differences in patient characteristics, including degree and nature of cardiomyopathy, or differences in metabolic background of the patients. It is also been suggested that the complexity of using the non-metabolizable substrates in PET scanning is not fully resolved [9].

An alternative and classic approach to estimate metabolic substrate utilization of the human heart is to establish arterio-venous gradients of substrates by sampling coronary sinus blood. Although this has been attempted in a meticulous study [10], the data on fatty acid uptake appear to be artificially low, presumably for methodological reasons and there is no estimation of triglyceride extraction. Based on the abundant data in rodents, and some evidence in humans [6], we hypothesized that failing human hearts would display a decrease in fatty acid utilization in preference for glucose in the fasted state i.e. when fatty acids should be preferentially used. We recruited well-matched heart failure, control and type 2 diabetes (T2DM) groups in whom we established coronary sinus sampling simultaneously with a constant intravenous infusion of [^2^H_2_]palmitate for specific quantification of fatty acid uptake and other metabolic substrates.

## Methods

### Study population

The study population consisted a group with heart failure (n = 9), T2DM (n = 5) and normal heart function (n = 10). The study was conducted prior to invasive electrophysiological investigation/treatment due to a case history of arrhythmia. Eight patients in the heart failure group had idiopathic dilated cardiomyopathy, and one had ischemic cardiomyopathy. Mean NYHA class of the heart failure group was 2.7±0.5. Oral administrations of β-adrenoreceptor blockers, were withheld at least 48 hours before the study. The control group consisted of ten patients with normal left ventricular function but a case history of either supra-ventricular tachycardia or paroxysmal atrial flutter. All participants showed normal sinus rhythm during investigation. The T2DM group consisted of three patients with heart failure and none of them were taking any oral anti-diabetic agent or insulin therapy.

All patients underwent a complete cardiac evaluation, including a history, physical examination and echocardiogram. The protocol was approved by the Oxfordshire Research Ethics Committee C and all participants gave written informed consent before the investigation.

### Investigations

All investigations were made following an 8-hour or overnight fast. Coronary sinus catheters were placed into coronary sinus via a femoral or a jugular vein approach aided by fluoroscopy. Arterial blood samples were from the femoral artery. All blood samples were taken before arrhythmia induction, ablation or pacemaker lead implant. No antiarrhythmic drugs or isoproterenol were administered before sampling. None of the patients had received heparin prior to, or during blood sampling. Patients received oxygen at 3–4 L/min through a face masks during the study.

A constant infusion of [^2^H_2_]palmitate (0.04 µmol/kg/min) dissolved in 4.5% human serum albumin was started 30 min prior to blood sampling. Three blood samples spaced 5 min were taken simultaneously from the arterial line and the coronary sinus and immediately transferred into heparinized tubes on ice. Plasma was separated within an hour.

### Chemical and stable isotope determinations

Quantification in plasma of non-esterified fatty acids, triacylglycerol, glycerol, glucose, pyruvate, lactate and 3-hydroxybutyrate as well as the determination of [^2^H_2_]palmitate enrichment was done exactly as described elsewhere [11]. Absolute non-esterified fatty acid extraction was calculated by knowing the proportion of palmitate to the total fatty acids. Blood gases were determined in triplicate (GEM Premier3000, Instrumentation Laboratory, Lexington, MA). BNP was analysed on a Siemens ADVIA Centaur (Siemens HealthcareDiagnostics, Frimley, UK) using an immunoassay with a coefficient of variation of 6.3%.

Substrate extraction was expressed in relation to O_2_ consumption using the Oxygen Extraction Ratio % (OER%) as described before [12] The potential O_2_ consumption resulting from uptake of each substrate was expressed as a percentage of total myocardial O_2_ consumption. The sum of OERs for the major myocardial substrates was greater than 100% (indicating that all substrates were not completely oxidized). To calculate the percentage contributions of different substrates to myocardial oxidative metabolism, these were adjusted to a total of 100%.

### Statistics

Data are presented as means±standard deviation using conventional methods (SPSS version 17) except for variables without normal distribution where median and range are shown. Skewness was tested using the Shapiro-Wilk normality test. Pearson correlation coefficients were calculated and for skewed variables Spearman Rank correlation coefficients were used. Statistical significances between groups were performed using Students unpaired t-test or Mann-Whitney U-test.

## Results

Age, body mass index, blood pressure and heart rate were comparable between the three groups (Table 1). A considerable elevation of plasma BNP together with lower echocardiographic-determined left ventricular ejection fraction (LVEF) verified the presence of heart failure. There was no significant difference in baseline plasma concentrations of metabolic substrates except higher glucose and lactate concentrations in patients in the T2DM group.

**Table 1:** Baseline description of heart failure, control and type 2 diabetes groups.

	Heart failure n = 9	Control n = 10	Type 2 diabetes n = 5
Gender (M/F)	7/2	4/6	5/0#
Age (years)	53±9	47±14	56±6
Body mass index (kg/m^2^)	30±7	27±5	28±3
Systolic BP (mmHg)	121±12	127±23	118±22
Diastolic BP (mmHg)	71±14	76±14	75±22
Heart rate (beats/min)	77±15	75±14	65±15
LVEF (%)	32±16$	59±5	49±21
BNP (pg/ml)	128 (22–432) *	16 (3–17)	41 (2–656)
Insulin (mU/l)	15±9	11±5	18±11
NEFA (µmol/l)	705±252	705±256	646±152
Triacylglycerol (mmol/l)	0.94±0.72	1.18±0.63	1.08±0.29
Glucose (mmol/l))	5.2±0.8†	5.3±0.4	7.0±0.7 *
Pyruvate (µmol/l)	67±34	46±20	56±28
Lactate (µmol/l)	524±235	546±140	802±298#
3-hydroxybutyrate (µmol/l)	232±208	177±149	112±71
Glycerol (µmol/l)	84±40	77±31	85±51

Values are mean±SD except for BNP which is shown as median (range).

# p<0.05 vs control,

* p<0.01 vs control,

$ p<0.001 vs control,

† p<0.01 vs DM using Student's t-test, except for BNP where Mann-Whitney U-test was used.

**Figure 1:**
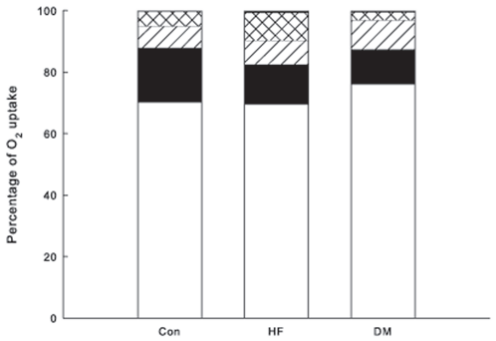
Contributions of different substrates to myocardial O_2_ consumption. Oxygen extraction ratios were summed and adjusted to a total of 100%. Lower, white bar: sum of non-esterified fatty acids and triacylglycerol (i.e. fatty acid oxidation); solid black, glucose; diagonal hatched, lactate; cross-hatched, 3-hydroxybutyrate; think solid line at top, pyruvate. Con, control group; HF, heart failure group; DM, Type 2 diabetes group.

Arterio-coronary sinus concentration differences of metabolites and fractional extractions were similar between the groups for all substrates except for lactate extraction, which was slightly higher in the T2DM group (Table 1 and 2). As there were no differences in oxygen extraction and little or no difference in proportional metabolite extraction between the groups, the OER% for the metabolites did not show any differences between the groups (Fig. 1).

**Table 2:** Fractional extraction (%) of non-esterified fatty acids (NEFA), triacylglycerol, glucose, pyruvate, lactate and 3-hydroxybutyrate across the heart in patients with heart failure, controls and type 2 diabetes.

	Heart failure n = 9	Control n = 10	Type 2 diabetes n = 5
NEFA	32±8	33±8	29±5
Triacylglycerol	1.8±3.5	2.8±2.4	3.5±2.0
Glucose	2.7±3.5	3.8±2.0	2.3±0.8
Pyruvate	23±31	28±15	40±37
Lactate	27±16	27±9	27±8
3-hydroxybutyrate	41±8	32±22	35±8

Values are mean±SD.

Arterial and coronary sinus tracer to trace ratio (TTR) of [^2^H_2_]palmitate were comparable between the three sampling occassions, which implies steady state. The TTR was consistently 15–20% lower in venous blood. The absolute non-esterified fatty acid plasma extraction assessed by isotopic tracer extraction was also similar between the three groups (140±40, 167±63, 148±61 µmol/l, heart failure, T2DM and control group, respectively (all ns)).

The fractional extraction of non-esterified fatty acids (r = 0.76, p<0.001), pyruvate (r = 0.82, p<0.001), lactate (r = 0.68, p<0.001) and 3-hydroxybutyrate (r = 0.98 p<0.001), were positively correlated with the arterial concentration for each substrate. In contrast, triacylglycerol and glucose extractions were unrelated to their respective arterial concentrations. Neither extractions of non-esterified fatty acids, [^2^H_2_]palmitate or of glucose showed any relationship to LVEF or plasma BNP levels.

## Discussion

We hypothesized to that failing hearts would display signs of reduced fatty acid uptake in favour of glucose, but there was no difference in the fractional extraction between fatty acid and glucose comparing healthy and failing hearts. As outlined in the Introduction, and also in a recent review [3], studies in animals provide a very consistent view that failing hearts have reduced fatty acid uptake whereas the few studies in humans are largely conflicting. It has also been suggested that the stage of heart failure is of importance as a switch towards glucose utilization is particularly easily observed in models of severe heart failure [3].

Our findings are in apparent contrast to those of Davila-Roman et al. [6] who found a shift towards glucose utilization in seven rather young patients with severe idiopathic dilated cardiomyopathy. Differences in clinical staging, the (in)comparability of control groups, and perhaps not least differences in technology could explain discrepancies between the studies. Of note, the heart failure and control groups were comparable for body mass index and fasting concentration of non-esterified fatty acids and blood glucose. Also, seemingly in contrast to Davila-Roman et al. [6], Wallhaus and colleagues [8] reported on the fatty acid and glucose utilization in considerably older patients undergoing significant improvements in heart function by carvedilol medication. The improvement (LVEF increasing from 25 to 37%) was linked to reduced fatty acid utilization but no apparent change in glucose uptake. However, Blain et al. performed direct measurements of metabolic substrate utilization in control and congestive heart failure patients by coronary sinus sampling showing that the respiratory quotient across the heart was close to 0.7 in both controls and patients [10] lending support to the notion of predominant fat oxidation in severe congestive heart disease.

We found a strong correlation between systemic non-esterified fatty acid concentrations and the uptake of fatty acids by the heart. Accordingly, background metabolic state may significantly affect the heart's preference for substrate, but in this study we were fortunate to observe extremely well-balanced background metabolic profiles between the groups.

The specific fatty acid uptake was quantified by isotope dilution technique and we were intrigued to find a 15–20% reduction in TTR of [^2^H_2_]palmitate/palmitate across the heart. This can only be the consequence of a dilution but the exact origin of the diluting fatty acids is unclear. A likely source is palmitate release from the epicardial adipose tissue. Epicardial fat may directly modulate the myocardium throughout vasocrine or/and paracrine pathways. The absence of fascial boundaries between the myocardium and the epicardial fat, supports the hypothesis of direct interconnection between the epicardial fat and the myocardium. The epicardial fat and the myocardium share the same microcirculation, including venous and arterial circulation [13].

Limitations of this study are the absence of blood flow measurements as well as substrate oxidation measurements. It would also have been of significant interest to observe the substrate utilization changes in response to insulin stimulation.

Although this study does not settle the issue of whether altered metabolic substrate utilization could be a cause or an adaptive consequence in heart failure it shows that the human moderately failing heart is not clearly prone to glucose utilization when this is quantified by direct methods, and it underscores the importance of robust methodologies to investigate the problem. There are now active research programs to develop therapies to interfere with fatty acid utilization in the failing heart [14], largely based on animal experiments, and it cannot be ruled out that humans are different.
